# Design and evaluation of a novel expandable gastroretentive film for sustained Metoprolol release

**DOI:** 10.1371/journal.pone.0345598

**Published:** 2026-04-01

**Authors:** Mustafa Musa Khalaf, Oday Sajad, Rana M. F. Sammour, Bazigha K. Abdul Rasool

**Affiliations:** 1 Department of Pharmaceutics, College of Pharmacy, University of Basrah, Basrah, Iraq; 2 Department of Pharmaceutics, College of Pharmacy, University of Misan, Misan, Iraq; 3 Pharmaceutical Sciences Department, College of Pharmacy, Dubai Medical University, Dubai, United Arab Emirates; Strides Pharma Science Limited, UNITED STATES MINOR OUTLYING ISLANDS

## Abstract

Gastroretentive drug delivery systems (GRDDS) are designed to prolong gastric residence time and improve drug bioavailability. Among these, swelling-based expandable films offer the dual advantage of increased gastric retention and controlled drug release. Metoprolol tartrate, a β1-receptor blocker with a short half-life and low oral bioavailability, was selected as the model drug. This study aimed to develop and evaluate a novel swelling-based expandable gastroretentive film of Metoprolol using a 4 × 4 full factorial design to systematically investigate the effects of HPMC and Carbopol 934 concentrations. Sixteen formulations (F1–F16) were prepared by solvent casting and swelling index (SI) and *in vitro* drug release were chosen as response variables. The optimized film (F14) exhibited a high SI and sustained drug release, delivering ≈86% of Metoprolol over 12 hours. Kinetic modeling demonstrated first-order and Higuchi release patterns with a non-Fickian mechanism, while model-independent parameters (AIC, MSC, similarity factors) supported these findings. Characterization studies confirmed the robustness of F14: FTIR and XRD revealed no drug–polymer interaction and an amorphous drug state, while SEM showed a uniform surface with interconnected pores. Mechanical testing confirmed desirable tensile strength and flexibility. Radiographic evaluation in rabbits demonstrated that F14 expanded and remained in the stomach for up to 12 hours, validating its gastroretentive capacity. Furthermore, short-term stability testing under accelerated ICH conditions confirmed the film’s physical and chemical stability and sustained-release performance for 6 weeks. In conclusion, the optimized expandable film (F14) combined swelling, gastric retention, and sustained drug release, highlighting its promise as a gastroretentive platform to enhance Metoprolol bioavailability and efficacy.

## Introduction

Oral drug delivery systems (DDSs) remain the most widely used approach in human medicine due to their convenience, versatility in formulation, cost-effectiveness, minimal storage requirements, and enhanced patient adherence. Despite these advantages, DDSs face challenges arising from physiological parameters, such as the complexity of the gastrointestinal tract (GIT), variable gastric pH, gastric retention time, surface area, and enzymatic activity, all of which can limit drug bioavailability [1,2]. Patients may also encounter issues such as incomplete drug release, reduced efficacy, frequent dosing, and insufficient gastric residence time.

To address these limitations, research over the past three decades has advanced the development of gastroretentive drug delivery systems (GRDDSs), particularly expandable platforms with distinct therapeutic advantages [3]. Compared with conventional oral formulations, GRDDSs enhance the bioavailability of drugs with a narrow absorption window in the upper GIT or those prone to degradation and reduced solubility at higher pH values. They are particularly suitable for drugs with short half-lives or those absorbed primarily in the upper intestine [4,5]. By prolonging gastric residence, GRDDSs enable controlled and continuous release of drugs at the absorption site, thereby maintaining steady therapeutic levels, optimizing dosing schedules, and reducing adverse effects [6–8].

Several GRDDS designs have been explored, including floating, swelling, and bioadhesive systems. However, their efficiency is influenced by formulation-related variables (polymer type, drug loading, excipients, film thickness), physiological conditions (gastric pH, motility, gastric emptying, fed or fasted state), and patient-related factors [9]. Among these platforms, expandable films offer distinct advantages. Their thin, flexible, and lightweight structure promotes gastric retention and minimizes the risk of dose dumping or obstruction. In addition, their relatively large surface area facilitates uniform drug release and improved control over release kinetics [10]. Compared with other GRDDS approaches, expandable films provide superior patient compliance, ease of administration, and adaptability in formulation design, making them an attractive option for gastroretentive drug delivery.

Metoprolol tartrate, a widely prescribed β-blocker, exemplifies the need for such systems. With a short elimination half-life (3–7 h), it requires frequent administration, which may compromise patient compliance and cause fluctuations in plasma concentration. Its moderate oral bioavailability (40–50%) is further reduced by extensive first-pass metabolism and variable GIT absorption [11]. Since absorption predominantly occurs in the upper intestine, prolonging gastric residence time provides an opportunity to enhance absorption and maintain consistent plasma levels.

Physicochemically, metoprolol tartrate is a crystalline white powder, highly soluble in water, soluble in methanol, and freely soluble in ethanol. It has a molecular weight of 684.8 g/mol, a water solubility of 0.402 mg/mL, a logP of 1.8, and a pKa of 9.7 [11]. Although several studies have investigated metoprolol floating tablets [12], no prior work has reported an expandable gastroretentive floating film formulation.

The present study introduces the first metoprolol-loaded expandable GR film designed to overcome the limitations of conventional GRDDS platforms. The formulation, prepared using HPMC K100M and Carbopol 934 as swellable polymers, was systematically evaluated for swelling behavior, *in vitro* drug release, and key physicochemical properties. *In vivo* testing in rabbits was conducted to assess gastroretentive performance and pharmacokinetic enhancement. Additionally, accelerated ICH stability testing confirmed that the developed film retained its physical integrity, chemical stability, and sustained-release performance for up to six weeks.

## Materials and methods

### Materials

Sama Alfayhaa Pharmaceutical Industries in Iraq donated Metoprolol tartrate. The polymers HPMC K100M and Carbopol 934 were purchased from Shanghai Ruizheng Chemical Technology Co., Ltd, China. Glycerin was obtained from the Central Drug House Ltd, India, and Ethanol absolute from EMSURE®, Germany. All the chemicals and reagents used were of analytical grade.

### Formulation and optimization of metoprolol films

#### Formulation design.

A 4 × 4 full factorial design was employed to investigate the effects of the independent variables, HPMC and Carbopol concentrations, on the dependent variables, swelling index (SI) and in vitro drug release (Table 1). These polymers were selected as key formulation components due to their critical influence on film performance. HPMC imparts film-forming ability, swellability, and controlled drug release through gel formation, whereas Carbopol enhances swelling and mucoadhesion, thereby supporting gastric retention. The factorial design enabled evaluation of both individual and interactive effects of these polymers, facilitating optimization of the formulation for sustained release and gastroretention. In total, 16 formulations were generated based on the design.

**Table 1:** Full factorial design based on independent and dependent variables.

Independent Variables	Levels			
HPMC (mg)	250	300	400	500
Cabopol 934 (mg)	100	150	200	250
**Dependent Variables**	**Required goals**			
Swelling index (%)	Maximum			
Drug release (%/h)	Maximum			

#### Preparation of GR films.

Metoprolol tartrate GR films were prepared by the solvent-casting method [13]. Accurately weighed quantities of HPMC and Carbopol 934P were dispersed in distilled water under continuous stirring until a homogeneous polymer solution was obtained. The required amount of Metoprolol tartrate was dissolved in a minimal volume of distilled water, then slowly added to the polymer solution with continuous stirring to ensure uniform mixing. Plasticizer (PEG 400/glycerol) was incorporated to improve film flexibility. The final solution was stirred and sonicated to remove entrapped air bubbles before being cast into levelled glass Petri dishes. Films were dried at 40 °C until formation, carefully peeled off, cut to a uniform size (2 × 2 cm), and stored in a desiccator for further evaluation.

All 16 formulations were prepared as listed in Table 2 and evaluated, with the formulation showing the best SI and *in vitro* release selected for further characterization.

**Table 2:** Composition of the prepared GR films (F1–F16) showing the varying concentrations of HPMC and Carbopol 934P used in the factorial design.

Formulations	Carbopol: HPMC (w/w)	HPMC (mg)	Carbopol 934 (mg)
F1	1.5:3	300	150
F2	2.5:5	500	250
F3	2:5	500	200
F4	2:3	300	200
F5	1:2.5	250	100
F6	2.5:2.5	250	250
F7	2.5:4	400	250
F8	1.5:2.5	250	150
F9	1:4	400	100
F10	1.5:4	400	150
F11	2.5:3	300	250
F12	1:5	500	100
F13	1:3	300	100
F14	2:4	400	200
F15	1.5:5	500	150
F16	2:2.5	250	200

All formulas contain Metoprolol (50 mg/film), ethanol 95% v/v (100 ml), glycerin (2 ml), and Ethyl Cellulose (100 mg).

### Optimization of the metoprolol films

**Swelling index:** The SI test is used to determine the degree of fluid uptake by the prepared film. The test was conducted using a 0.1N HCl solution at ambient room temperature. The initial weight of the film was measured at zero time, and the film was then placed over a cotton piece thoroughly wet with 0.1N HCl. The film’s weight was measured at intervals until a constant weight was observed [13].

The swelling index was calculated using the following equation:


$$SW=\frac{Wt-W_{0}}{W_{0}}$$ (1)

Where:

Wt: weight at time t

W_0_: initial weight

***In vitro* drug release:** The *in vitro* dissolution of the prepared films was carried out in a type II dissolution apparatus. The Metoprolol gastroretentive films were immersed in 900 ml of the simulated gastric dissolution medium (simulated gastric fluid made up of CaCl_2_, 0.1N HCl, and distilled water) at 37 ± 0.5 ^0^C and stirred at 50 rpm. At predetermined time intervals, a 5 ml solution of the solvent was extracted, filtered, and analyzed using UV Spectroscopy at a wavelength of 221 nm. The withdrawn samples were replaced with an equal volume of the simulated gastric fluid [14].

#### Kinetic modeling.

DDSolver, an add-in software integrated with Microsoft Excel, was utilized to forecast several dissolution models, including but not limited to zero-order, first-order, Higuchi, and Korsmeyer-Peppas. These models have been widely employed in drug release prediction. The correlation coefficient (R^2^) and rate constant (K) values for each model were computed by the software. Moreover, the release exponent (n) in the Korsmeyer-Peppas model was determined to describe the drug release mechanism from the film [15]. The zero-order model assumes a concentration-independent release mechanism, in which the drug is gradually released from the formulation. On the other hand, the first-order kinetic model elucidates drug release as a function of concentration. The Higuchi model characterizes the release of the drug from the matrix dosage form [16].

The equations for the previously mentioned models are as follows:

For the zero-order model:


$$F=\;k_{\circ \;}\times t$$ (2)

Where:

F: Fraction of the released drug at time t.

k_0_: Release constant of zero-order model.

For the first-order model


$$\text{log}C=logC_{\circ }-\;\frac{k_{t}}{\text{2}.\text{3}\text{0}\text{3}}$$ (3)

Where:

C_0_: Initial concentration of the drug.

C: Concentration of the drug at time t.

K: Release rate constant of the first-order model.

For the Higuchi model


$$Q=\;k_{H}\times \;t^{\text{1}/\text{2}}$$ (4)

Where:

Q: Amount of drug released in time t per unit of area.

K_H_: Higuchi constant

For the Korsmeyer-Peppas model


$$\frac{M_{t}}{M_{\infty }}=\;k_{m}t^{n}$$ (5)

Where:

Mt/M_∞_: Fraction of drug released at time t.

Km: Release rate constant.

n: Release exponent.

#### Dissolution profile comparison

The similarity factor (f2) was used to compare the in vitro drug release profiles of the prepared gastroretentive films with those of the marketed reference product (Metoprolol Tartrate 50 mg, BRISTOL®). The analysis was performed according to the FDA and EMA guidelines, which recommend using f2 as the logarithmic reciprocal square root transformation of the sum of squared differences between the test and reference profiles [17].

In this study, cumulative drug release data obtained in simulated gastric fluid (pH 1.2) at predetermined time points were used for the calculations. According to regulatory standards, an f2 value between 50 and 100 indicates similarity between two dissolution profiles. The calculated f2 values confirmed that the optimized formulation exhibited a release pattern comparable to the marketed product.

##### Selection of the optimized formulation.

An overlay contour plot was generated in Minitab® 17 (Response Optimizer) from the final regression models for the two critical responses, swelling index (SI, %) and drug released at 12 h (%), across the HPMC (mg)–Carbopol 934 (mg) factor space to identify the multi-response feasible region and the optimal set point. Consistent with the goals in Table 1, both responses were set to maximize in the multi-response optimization using the Derringer–Suich composite desirability (D, 0–1). The overlay highlights the region where both responses are simultaneously favorable, and the numerical optimum was selected by maximizing D.

#### Characterization of the optimized formula

##### Film thickness and weight uniformity.

The thickness of each film was measured at different points (n = 6) using a micro vernier. The mean film thickness ± SD was determined.

The film's weight uniformity was assessed by cutting three pieces (2 × 2 cm) from different regions of each film batch and weighing them individually on a calibrated Sartorius analytical balance (Entris® II, Sartorius AG, Göttingen, Germany). The mean weight and percentage variation were calculated to evaluate uniformity. The percentage weight variation was determined according to the formula:


% Weight Variation = Standard Deviation/ Mean Weight × 100 (6)

##### Folding endurance.

The folding endurance of the film was measured by repeatedly folding it at the same point until it broke or the folding number reached 200 [13].

##### Expansion time.

The expansion time was conducted using the same conditions as the *in vitro* drug release test. The optimized film was folded and placed in a capsule (Capsule size used was 000). The film's length was measured at 10 minutes and again at 15 minutes.

##### Tensile strength.

Tensile strength is the highest force that a material can withstand before breaking. The test was conducted using the texture analyzer testing machine (TA.XTplus, Stable Micro Systems, Godalming, Surrey, UK). The film was placed between the tensile grips probes. One grip was fixed to the platform, and the other moved up, and the weights were added gradually until the film broke. Tensile strength was measured with a load cell of 5 kg, pre-test speed of 1 mm/sec, test speed of 2 mm/sec, post-test speed of 10 mm/sec, and trigger force of 5 g [18]. The formula for determining the tensile strength is:


$$TS=\;\frac{F}{H}$$ (7)

Where:

TS: Tensile Stress, F: Force, H: Surface Area. The unit is: Newton per meter (N. × m^2^)

##### Film burst.

The film burst serves as a gauge for the material's resistance to rupturing. It assesses the behavior of a film when it is stretched beyond the limit at which it deforms or ruptures. The measurement of the film burst was done using a texture analyzer. A film-supporting rig (HDP/FSR) probe was used with a 5 kg load cell; the film was mounted on a heavy platform, and the ball probe was allowed to penetrate the film. The test was performed with a pre-test speed of 2 mm/sec, a test speed of 1 mm/sec, and a post-test speed of 10 mm/sec, with a trigger force of 5 g [18].

##### FT-IR spectroscopy.

IRAffinity-1S FT-IR spectroscopy (Shimadzu, Japan) was utilized as an experimental technique to ascertain the presence of chemical incompatibility between the drug and excipients in the ultimate formulation. The experimental procedure, with modifications, involved analyzing the pure medication, the physical mixture in a 1:1 ratio, and the optimized formula. The physical mixture was prepared by blending an equal quantity of the drug and the polymers. A KBr disc was used, scanned at 400−4000 cm^-1^, with a resolution of 4 cm^-1^ [19].

##### X-Ray diffraction.

The crystallinity of the drug and the optimized formula were assessed using X-ray diffraction (XRD) analysis, employing a Shimadzu- Japan X-ray diffractometer. The samples were analyzed at 25 °C and exposed to radiation at a voltage of 40kV and a current of 20 mA; the angle range of scanning was 10–80° [19].

##### Scanning electron microscopy (SEM).

The film underwent analysis using ZEISS scanning electron microscopy (SEM) (Germany) to investigate its surface morphology. The instrument employed secondary electron imaging; the film was mounted on a metal plate inside the device, and the film was observed at 10 kV and 50 kV magnification [19].

##### Differential scanning calorimetry.

The molecular structure of the medication was analyzed using DSC-60 plus differential scanning calorimetry (DSC), Shimadzu, Japan. The analysis was done on the drug powder, physical mixture (1:1), and optimized formula. The samples were kept in an aluminium pan, and the scanning rate was 10 °C/min. The temperature range for the drug was 20 °C to 260 °C, and for the physical mixture and polymers, it was 50 °C to 550 °C. Nitrogen was used as a purge gas [19].

##### Film integrity.

Film integrity (F14) was evaluated by immersing the films in 0.1 N HCl and visually inspecting them at predetermined time intervals (0, 4, 8, and 12 h). The beakers were maintained in a thermostatically controlled water bath at 37 ± 0.5 °C to mimic physiological temperature, with occasional gentle agitation to ensure uniform contact of the medium with the film surface.

At each point, the films were examined for signs of swelling, cracking, erosion, or disintegration to assess their structural stability under simulated gastric conditions.

#### Radiographic evaluation

All animal experiments were conducted in accordance with internationally accepted principles for the care and use of laboratory animals, following the NIH Guide for the Care and Use of Laboratory Animals (8th edition, 2011) [20]. The study protocol was reviewed and approved by the Ethical Committee at the College of Pharmacy, University of Misan, Misan, Iraq (Approval Reference: EA 315, Date: 10/09/2024). Radiological studies were performed at the Radiological Department of Misan Veterinary Hospital, Misan, Iraq.

Four healthy male New Zealand White (NZW) rabbits (average weight 1.0–1.4 kg) were selected for the in vivo evaluation. Animals were housed individually in cages under controlled conditions (25 ± 1 °C) and acclimatized for 7 days. They were free of gastrointestinal symptoms, maintained on a standard diet, and fasted for 12 hours before the study, with free access to water.

The optimized metoprolol-loaded expandable film (F14) was incorporated with 30% w/w barium sulfate as a radiopaque marker to enable visualization. Films were administered orally with adequate water. Radiographic images were taken at predetermined intervals (0, 6, and 12 h) using a TOSHIBA™ X-ray machine, with both lateral and frontal views captured. The baseline (0 h) radiograph confirmed the absence of radiopaque material prior to dosing. During imaging, animals were positioned securely to minimize movement.

For animal welfare, sedation and analgesia were applied: Xylazine (2–4 mg/kg, IM) and Ketamine (15–20 mg/kg, IM). This ensured minimal stress and humane handling during radiographic procedures.

No animals were sacrificed in this study. As the procedures were non-terminal and limited to radiographic imaging under sedation, all animals recovered uneventfully and were returned to the animal facility after the completion of the study. The experimental protocol, including the decision not to sacrifice animals, was reviewed and approved in accordance with the NIH Guide for the Care and Use of Laboratory Animals (8th edition, 2011).

A summary of the animal model, housing, treatment, and imaging schedule is presented in Table 3.

**Table 3:** Experimental design of the *in vivo* radiographic study.

Parameter	Details
Animal model	Healthy NZW male rabbits
Number of animals	Four
Weight range	1.0–1.4 kg
Housing conditions	25 ± 1 °C, individual cages, 7-day acclimatization
Feeding	Standard diet; fasted 12 h before study (water allowed)
Formulation administered	Optimized film (F14) containing 30% w/w barium sulfate
Route of F14 administration	Oral, with adequate water
Analgesia	Xylazine (300 mg/ml, 30 ml vial), dose (2–4) mg/kg, IM injection.
Anesthesia	Ketamine (100 mg/ml, 5 ml vial), dose (15–20) mg/kg, IM injection
Imaging equipment	TOSHIBA™ X-ray machine
Imaging schedule	0, 6, and 12 h post-administration
Observation	Gastric retention and expansion confirmed via lateral and frontal views

#### Short-term stability study

Short-term stability testing of the optimized GR film (F14) was performed for 6 weeks following ICH Q1A(R2) guidelines under accelerated conditions. Samples were stored in a controlled stability chamber (Memmert HCP 108 Climate Chamber, Germany) with continuous monitoring and calibration to ensure compliance with ICH standards. Films (2 × 2 cm) were stored in packed (aluminium foil sachets and amber HDPE jars) and unpacked (open petri dishes) forms at 40 ± 2 °C and 75 ± 5% RH. Samples were withdrawn at 0, 1, 3, and 6 weeks (n = 6) and evaluated for appearance, thickness, weight uniformity, moisture content, tensile strength, swelling index, and *in-vitro* release in 0.1 N HCl (0–12 h, USP II, 50 rpm). FTIR was used to check for solid-state changes at 0 and 6 weeks [19].

#### Statistical analysis & software

Minitab® 17 was used to build a Full-Factorial Design, and a One-Way analysis of variance (ANOVA) was performed. Moreover, a Microsoft Excel Add-In called DDSolver was used for both model-dependent and model-independent drug release. All evaluation test results are expressed as mean ± SD (n = 3). Differences were considered statistically significant at p < 0.05. 3D Surface Plot of Response and 2D Contour Plot of Response Figures were generated using Python® 3.11 with the Matplotlib® (v3.8) and NumPy® (v1.26) libraries.

## Results and discussion

### Formulation of metoprolol GR film

The expandable gastroretentive (GR) film strategy provides several distinct advantages compared to other GR platforms, such as floating tablets, swelling devices, or mucoadhesive systems. These films are inherently thin, flexible, and lightweight, which facilitates prolonged gastric retention while minimizing the risks of dose dumping, obstruction, or patient discomfort. Their relatively large surface area promotes more uniform drug release and improved modulation of release kinetics [3]. Moreover, expandable films offer greater ease of administration, enhanced patient compliance, and versatility in formulation design, making them particularly attractive for chronic therapies that require sustained release. Taken together, these attributes justified selecting the expandable film platform as the most suitable approach for sustained metoprolol delivery.

HPMC and Carbopol 934 were selected as key formulation variables because they directly influence film swelling, structural integrity, and drug release. HPMC imparts film-forming capability, swellability, and controlled release via gel formation, whereas Carbopol enhances swelling and mucoadhesion, promoting gastric retention [21].

### Optimization of metoprolol films

In our study, a 4x4 Full Factorial Design was employed to systematically investigate the effects of varying concentrations of HPMC and Carbopol 934 on two dependent variables: the swelling index (SI) and *in vitro* drug release. These two response variables were selected because they directly reflect the intended functionality of an expandable gastroretentive drug delivery system. The Swelling Index (SI) is a critical determinant of gastric retention: a higher swelling capacity increases film size, which helps prevent premature passage through the pylorus and prolongs gastric residence time [22]. Conversely, *in vitro* drug release is crucial for ensuring the sustained and predictable delivery of Metoprolol, thereby maintaining therapeutic plasma levels, minimizing dosing frequency, and enhancing patient adherence [23].

It was anticipated that varying the concentrations of HPMC and Carbopol would influence these responses: HPMC, as a hydrophilic swellable polymer, enhances water uptake and drug diffusion, while Carbopol contributes to matrix viscosity and mucoadhesion, retarding drug release and prolonging gastric retention. According to the factorial design, 16 formulations (F1–F16) were generated, enabling systematic evaluation of both the individual and interactive effects of these polymers on film performance.

#### Swelling index evaluation.

**Fig 1:**
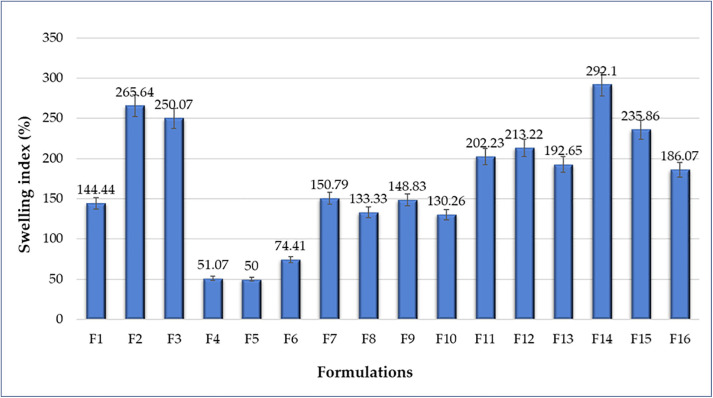
Swelling index (SI, %) of GR film formulations (F1–F16). Data are presented as mean ± SD (n = 3).

The SI test is crucial for the bioadhesive behavior of the prepared films [23]. The higher the SI, the greater the bioadhesion. The developed formulas showed a wide SI range, ranging from 50 to 292, as shown in Fig 1. According to the statistical studies, a significant difference (*p* < 0.05) was recognized between the formulations.

The formulas, F2, F3, F11, F12, F14 and F1F scored SI more than 200. The previously mentioned formulas were recognized for their high amount of HPMC (400 or 500 mg HPMC). HPMC showed a significant difference towards the SI (*p* < 0.05). The higher the polymer amount, the higher the SI. However, the results revealed that Carbopol didn't yield a statistically significant difference (p > 0.05). Among all the formulations, F14 exhibited the highest SI. This is due to the quantity of polymers used in this formulation, as both HPMC and Carbopol were high. The HPMC enhanced the bioadhesion of the films. Most of the studies concluded that the higher the HPMC, the higher the SI and hence the bioadhesion of the film, and the statistical analysis confirmed the same results as shown in Fig 3A [24]. Similarly, for Carbopol, as a cross-linking agent, it can retain more water in the film as its concentration increases, leading to higher bioadhesion [24].

**Fig 2:**
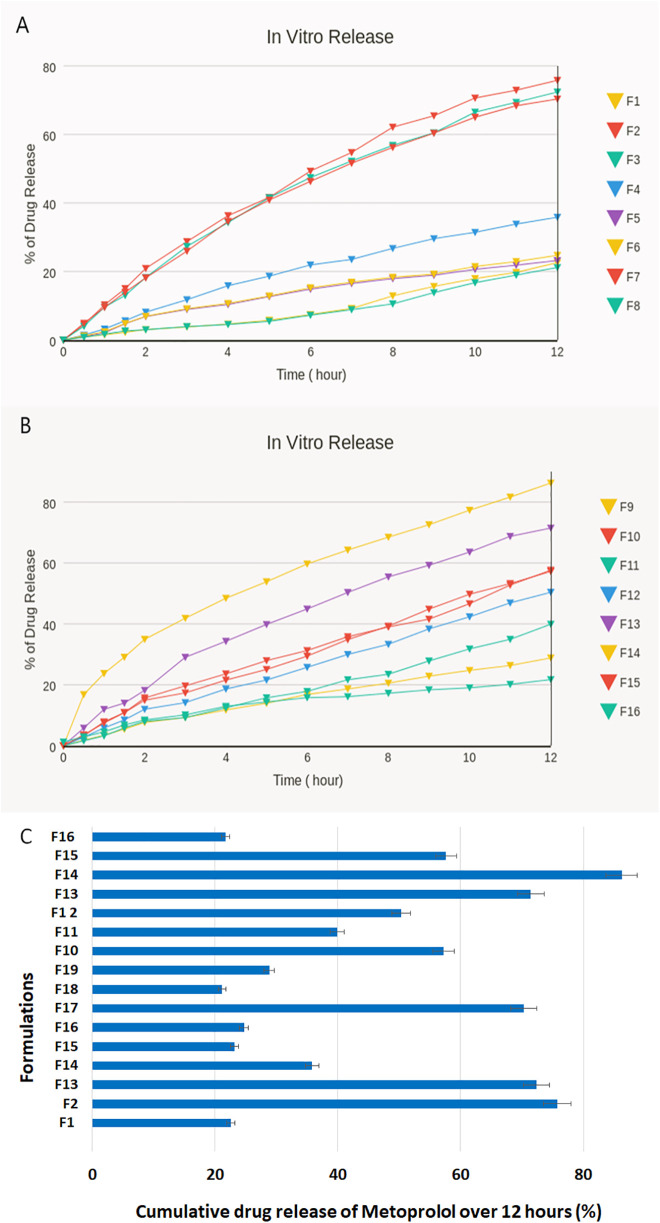
Percentage of cumulative drug release of the expandable GR films. (A) Formulations F1–F8, (B) Formulations F9–F16, and (C) Formulations (F1–F16) after 12 hours of dissolution run. Data are shown as mean ± SD (n = 3). Formulation F14 showed a statistically significant difference (p < 0.05) compared to other formulations.

#### Drug release studies.

Results showed that all formulations exhibited a gradual, controlled release profile without an initial burst effect. However, specific formulations demonstrated incomplete release within 12 hours, achieving only 30–70% of the total drug release (Fig 2). The following formulations: F2, F3, F7, F13, and F14 were among the formulations that showed the highest drug release. The quantity of polymers and their combination were crucial in affecting the drug release from the film matrix. The formulations that exhibited higher release had higher amounts of both polymers.

Comparing the drug release of the formulations, it was apparent that F14 had the highest release, reaching 86.24%. The HPMC amount significantly (*p* < 0.05) affected the drug release. At the same time, the Carbopol amount had an insignificant effect (p > 0.05). It was evident that the release rate decreased with increasing Carbopol concentration. These findings were reported by Jin-Wook Yoo et al. (2006) [25].

**Fig 3:**
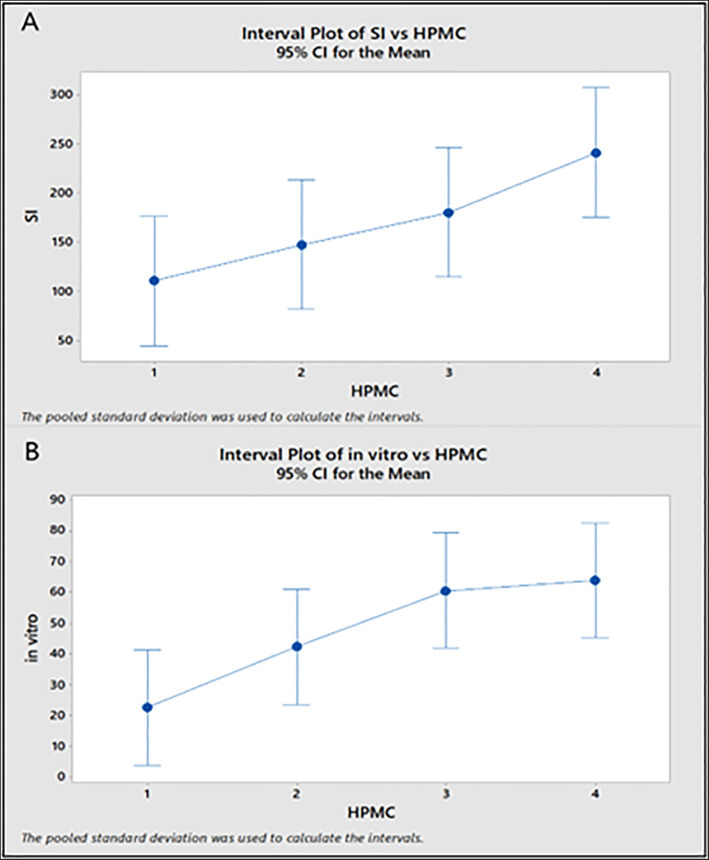
Interval plots by one-way ANOVA showing: A) swelling index (SI) vs. HPMC concentration, and B) in vitro drug release vs. HPMC concentration. Error bars represent 95% confidence intervals (CI).

**Fig 4:**
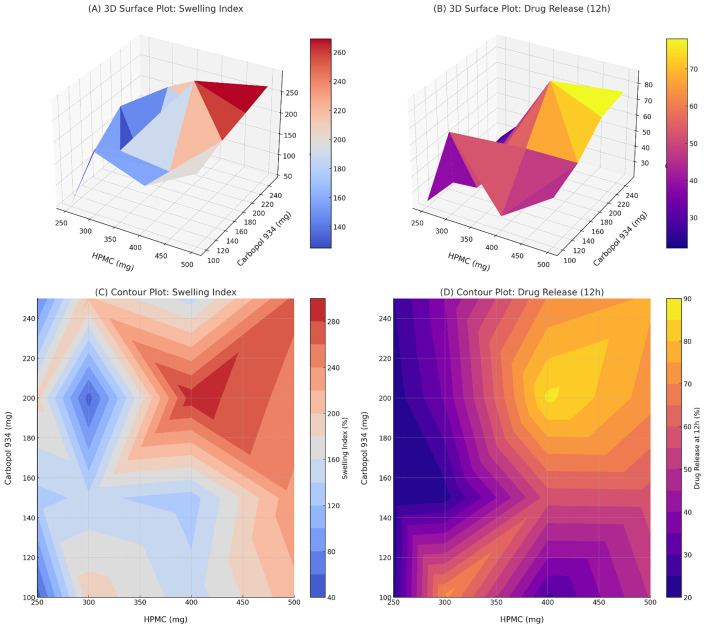
Optimization plots illustrate the effect of HPMC and Carbopol concentrations on formulation responses. (A) 3D surface plot of swelling index (%); (B) 3D surface plot of drug release at 12 h (%); (C) Contour plot of swelling index (%); and (D) Contour plot of drug release at 12 h (%).

Furthermore, the experimental design allowed us to evaluate how changes in polymer concentration affected responses and to identify the optimal formulation, as shown in Fig 3. The optimized film (F14) was thus developed based on a scientific understanding of the critical formulation parameters, rather than through trial and error. Fig 4 represents optimization plots illustrating the effect of HPMC and Carbopol concentrations on formulation responses.

Unlike conventional floating gastroretentive systems, the present formulation was designed as an expandable GR film. Its gastric retention is achieved through rapid hydration and swelling, which increases its size and prevents passage through the pylorus. Therefore, floating time studies were not performed, as buoyancy is not the retention mechanism in this system. The expandable behavior and gastric retention were instead confirmed through %SI measurements and further substantiated by the *in vivo* radiographic study, which directly visualized the prolonged gastric residence of the film.

### Kinetics modelling

The drug release kinetics of the prepared films were evaluated using the following mathematical models: zero-order, first-order, Higuchi, and Korsmeyer–Peppas [16]. The best-fitting model for each formulation was determined by the highest adjusted R² (Table 4). Application of the Korsmeyer–Peppas model indicated that all formulations followed a non-Fickian (anomalous) transport mechanism, since the *n* values were greater than 0.5. This finding suggests that drug release was governed by a combination of dissolved drug diffusion through the swollen polymeric matrix and polymer chain relaxation or erosion [26].

**Table 4:** Kinetics modeling following linear regression of *in vitro* drug release.

Modeling	Formulas	Parameters	R^2^ adj.	AIC	MSC	Modeling	Formulas	Parameters	R^2^ adj.	AIC	MSC
**Zero-order**	F1	K0 = 1.67	0.95	55.69	2.84	**First-order**	F1	K1 = 0.018	0.93	59.7	2.57
	F2	K0 = 7.91	0.95	93.25	2.76		F2	K1 = 0.116	0.99	46.37	5.89
	F3	K0 = 6.8	0.95	91.09	2.81		F3	K1 = 0.106	0.99	34.11	6.61
	F4	K0 = 3.23	0.97	58.36	3.6		F4	K1 = 0.039	0.99	35.58	5.12
	F5	K0 = 2.14	0.95	56.8	2.78		F5	K1 = 0.024	0.97	48.65	3.33
	F6	K0 = 2.22	0.96	54.72	3		F6	K1 = 0.025	0.98	45.39	3.63
	F7	K0 = 6.69	0.95	91.74	2.71		F7	K1 = 0.103	0.99	18.99	7.56
	F8	K0 = 1.55	0.95	54.08	2.77		F8	K1 = 0.017	0.93	57.64	2.54
	F9	K0 = 2.53	0.97	50.61	3.56		F9	K1 = 0.029	0.99	35.89	4.55
	F10	K0 = 4.94	0.98	63.75	4.06		F10	K1 = 0.065	0.98	61.35	4.22
	F11	K0 = 3.15	0.99	40.68	4.87		F11	K1 = 0.037	0.98	55.62	3.87
	F12	K0 = 4.27	0.99	49.95	4.74		F12	K1 = 0.054	0.99	53.44	4.51
	F13	K0 = 6.65	0.94	91.84	2.65		F13	K1 = 0.103	0.99	45.12	5.76
	F14	K0 = 8.34	0.77	116.91	1.01		F14	K1 = 0.16	0.96	89.64	2.83
	F15	K0 = 4.92	0.97	74.58	3.26		F15	K1 = 0.065	0.9896	59.9153	4.2457
	F16	K0 = 2.09	0.79	74.71	1.18		F16	K1 = 0.024	0.84	70.74	1.45
**Higuchi**	F1	kH = 4.54	0.74	81.44	1.12	**Korsmeyer-Peppas**	F1	kKP = 0.65 n = 1.42	0.98	35.73	4.17
	F2	kH = 20.52	0.94	97.26	2.49		F2	kKP = 11.21 n = 0.82	0.99	28.79	6.56
	F3	kH = 19.37	0.93	96.43	2.45		F3	kKP = 10.29 n = 0.79	0.99	44.65	4.59
	F4	kH = 9.14	0.91	80.09	2.15		F4	kKP = 4.76 n = 0.82	0.99	37.61	4.98
	F5	kH = 6.09	0.93	61.83	2.45		F5	kKP = 3.72 n = 0.74	0.99	31.43	4.47
	F6	kH = 6.31	0.93	63.77	2.4		F6	kKP = 3.72 n = 0.76	0.99	26.69	4.87
	F7	kH = 19.08	0.94	94.63	2.52		F7	kKP = 10.9 n = 0.79	0.99	38.68	5.05
	F8	kH = 4.23	0.74	78.68	1.13		F8	kKP = 0.61 n = 1.42	0.98	38.98	3.78
	F9	kH = 7.18	0.92	70.03	2.27		F9	kKP = 3.88 n = 0.80	0.99	11.39	6.18
	F10	kH = 13.93	0.9	94.11	2.04		F10	kKP = 6.66 n = 0.86	0.99	49.88	4.99
	F11	kH = 8.79	0.85	89.13	1.64		F11	kKP = 2.97 n = 1.02	0.99	42.05	4.77
	F12	kH = 11.99	0.89	92.08	1.93		F12	kKP = 5.26 n = 0.90	0.99	39	5.47
	F13	kH = 19	0.95	91.65	2.66		F13	kKP = 11.76 n = 0.74	0.99	37.92	5.01
	F14	kH = 24.44	0.99	30.72	6.75		F14	kKP = 23.94 n = 0.5	0.99	5.42	6.87
	F15	kH = 13.96	0.92	89.1	2.29		F15	kKP = 7.78 n = 0.78	0.99	51.74	4.79
	F16	kH = 6.11	0.98	31.74	4.05		F16	kKP = 5.61 n = 0.54	0.99	29.53	4.2

Among the kinetic models examined, the first-order model consistently showed the highest correlation, particularly for the optimized film (F14), indicating that drug release was concentration-dependent and the release rate declined as the drug concentration in the matrix decreased.

The Higuchi model also provided good fits for most formulations, including F14, supporting the conclusion that diffusion through a porous polymeric structure was a significant release mechanism [27]. In contrast, the zero-order model showed lower R² values, suggesting that a constant release rate was not the dominant mechanism in this system.

The composition of the films can explain the observed kinetic behavior. HPMC, being hydrophilic, rapidly hydrates and swells in the dissolution medium, forming a gel layer that both stabilizes the film structure and creates diffusional pathways as the drug dissolves and escapes. The resulting porous matrix facilitates sustained release through diffusion. Carbopol complements this effect by forming a viscous gel layer that slows water uptake and modulates polymer solubility, thereby retarding drug release. Additionally, ethyl cellulose contributes a hydrophobic barrier, further prolonging the release process [28]. Taken together, these results confirm that F14 and the other formulations predominantly follow first-order and Higuchi release kinetics, with diffusion as the primary mechanism supported by swelling and erosion of the polymer matrix.

To evaluate the mechanism of drug release from the prepared films, the release data were fitted to the following mathematical models: zero-order, first-order, Higuchi, and Korsmeyer–Peppas [26]. The adequacy of each model was assessed using correlation coefficients (R²) and additional statistical parameters, including the Akaike Information Criterion (AIC) and the Model Selection Criterion (MSC). In this context, the most precise model corresponds to the lowest AIC value, whereas the highest MSC indicates the best model fit [15].

**Fig 5:**
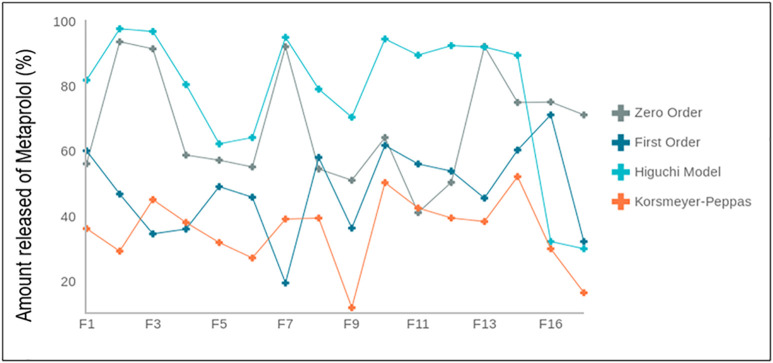
Akaike information criterion (AIC) of the formulation F1-F16, calculated using different kinetic models.

Fig 5 presents the release profiles fitted to the different models, while Table 3 summarizes the corresponding kinetic parameters. The linearity and R² values provide insight into the drug release mechanism. For example, a good fit with the first-order model indicates a concentration-dependent release process, whereas a good fit with the Higuchi model suggests diffusion through a polymeric matrix. The Korsmeyer–Peppas model further clarifies the mechanism: a release exponent (n) > 0.5 indicates non-Fickian (anomalous) transport, indicating that both diffusion and polymer relaxation/erosion contribute to drug release [15].

**Fig 6:**
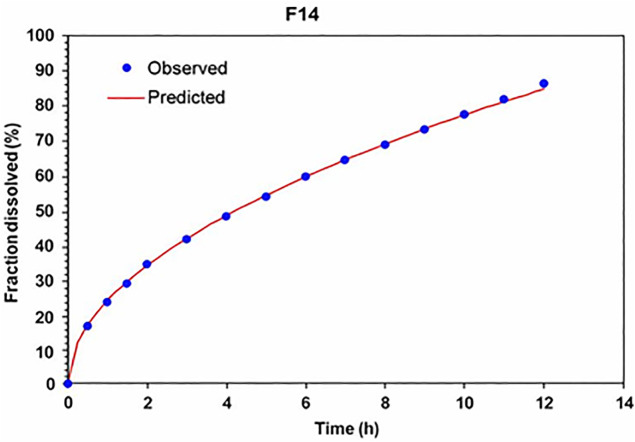
The correlation between the metoprolol amount released from the F14 formula versus the predicted amount of metoprolol by the Higuchi model.

According to the AIC results, the Korsmeyer–Peppas model showed the best fit, followed by the first-order model, supporting the predominance of diffusion- and relaxation-controlled mechanisms [15]. For the optimized film (F14), both the Higuchi and Korsmeyer–Peppas models provided the best fit, as also confirmed by MSC values (Table 4). In addition, Fig 6 shows a strong correlation between the observed and predicted drug release values for formulation F14 under the Higuchi model, indicating a steady release profile without burst effects. This graphical confirmation presents how well the experimental data aligns with the theoretical prediction.

This behavior can be attributed to the polymer composition of films. HPMC rapidly swells to form a porous gel matrix, facilitating water uptake and creating diffusion pathways, while Carbopol forms a viscous barrier that slows water penetration and retards release. Ethyl cellulose, due to its hydrophobic nature, further contributes to prolonged release [29]. Together, these polymers establish a release pattern characterized by controlled diffusion and matrix erosion.

Such detailed kinetic modeling ensures transparency, reproducibility, and clear comparison among formulations, which are critical for formulation optimization and align with best practices reported in the literature. Moreover, advanced tools such as DD-Solver were employed for model fitting and to assist in selecting the optimized formulation, thereby supporting the application of quality-by-design (QbD) principles in dosage form development [8].

### Similarity factor analysis (f2)

The utilization of the similarity factor (f2) as a valuable means to assess and compare dissolution profiles, thus aiding in selecting the optimized formulation. Results from the f2 analysis confirmed that the formulated formula obtained significantly different results than the chosen reference (Metoprolol Tartrate 50 mg, BRISTOL). The f2 value was 10.7, below 50, indicating dissimilarity between the formulated film and the reference.

### Selection of the optimized formulation

**Fig 7:**
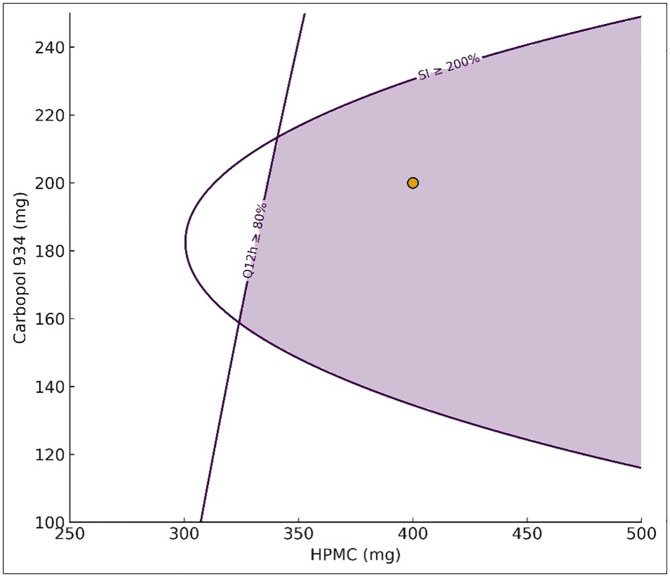
Overlay contour plot of the HPMC (mg)-Carbopol 934 (mg) factor space showing the multi-response design space. Contours correspond to swelling index (SI, %) and % released at 12 h (Q12h); the shaded region denotes the area that simultaneously satisfies SI ≥ 200% and Q12h ≥ 80%. The marker indicates the optimized batch (F14) at HPMC 400 mg; Carbopol 200 mg, which lies within the feasible region and achieved Q12h = 86.24% (composite desirability D = 1.00).

The overlay (Fig 7) delineated a contiguous feasible region in the HPMC–Carbopol plane. In agreement with the observed trends, higher HPMC increased drug release, whereas increasing Carbopol tended to reduce it; the feasible region was located at higher HPMC with mid Carbopol levels. The optimized batch (F14) at HPMC 400 mg and Carbopol 200 mg fell within this region. It exhibited the highest 12 h release (Q12h = 86.24%) together with a high SI (%), justifying its selection for complete characterization. Composite desirability (D = 1) was maximized at this set-point [4].

### Characterization of the optimized formula

#### Film thickness and weight uniformity.

The mean thickness of the optimized films was (0.5 ± 0.02 mm). The thickness, as compared with another study, was acceptable concerning different concentrations of the used polymers. Using HPMC increases the film thickness [28].

Additionally, the prepared films exhibited good weight uniformity. The mean weight of the samples ranged from 48.7 ± 1.3 mg to 52.4 ± 1.6 mg, with percentage variation less than 3% across all batches, confirming excellent reproducibility. These results demonstrate that the solvent-casting method provided consistent film thickness and homogeneous drug–polymer distribution, in agreement with previously reported findings on polymeric drug delivery films [30].

#### Folding endurance.

The optimized formula (F14) yielded a folding endurance value that exceeded 200 folds without breaking. This demonstrates excellent mechanical strength and flexibility, ensuring the film withstands repeated handling during manufacturing, packaging, and administration. Such durability minimizes the risk of brittleness or cracking during storage and use.

#### Expansion time.

Regarding the film's expansion, the unfolding and expansion occurred within 15 minutes, reaching a final length of 22 mm. This rapid expansion indicated prompt swelling and gastric unfolding, a critical property for GR systems, as it helps the dosage form remain in the stomach for prolonged periods. The results obtained aligned with those reported in previous studies; for example, a study on starch/chitosan expandable films found that the film unfolded completely within 15 minutes when exposed to simulated gastric fluid [31].

#### Tensile strength.

Tensile strength is defined as the maximum stress a film can withstand before breaking, and it is calculated by dividing the maximum applied force by the product of the film’s width and thickness [31]. Crossing the design space, the optimized film F14 displayed self-supporting, non-brittle behavior with tensile strength profiles consistent with its composition: increasing HPMC proportion generally produced stronger, more cohesive matrices, whereas higher plasticizer content (e.g., PEG/glycerol) tended to lower tensile strength but increased the film’s flexibility and elongation, a well-described trade-off in cellulosic films. Carbopol contributed to the formulation's structural integrity by forming a network and hydrogen bonding with HPMC, thereby providing adequate resistance to tearing during unfolding and supporting gastric motility. The tensile strength of the optimized film (F14) was approximately 1.1 ± 0.05 kg, which is sufficient to withstand routine handling, packaging, and in-use unfolding, and encapsulation but intentionally lower than the tensile strength typically required for transdermal films (often >2 kg), as gastroretentive films need to unfold and expand rapidly in the gastric environment rather than remain rigid for extended dermal adhesion. These trends align with prior literature on expandable and mucoadhesive films, where plasticizer-induced TS reductions accompany gains in flexibility, and polymer network composition governs stiffness and robustness [27].

#### Film burst test.

**Fig 8:**
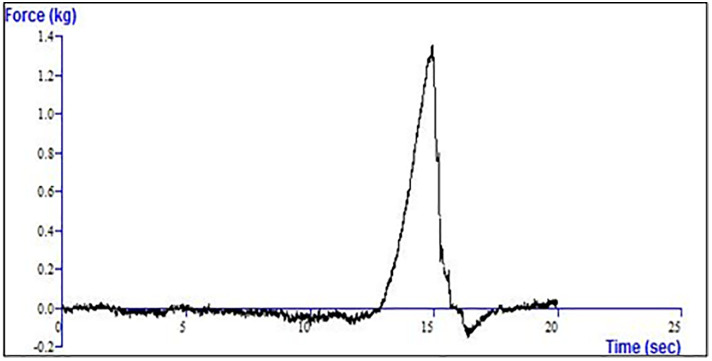
Film burst analysis of (F14) during tensile strength testing. The curve shows the application of force (kg) over time (sec), with a sharp peak indicating the maximum force required before film rupture.

Burst strength is a critical property that reflects the resistance of gastroretentive films to rupture under pressure and is influenced by both tensile strength and extensibility [31]. The burst test was carried out using the TA-XTPlus Texture Analyzer, applying force until the film ruptured. As shown in Fig 8, the optimized film (F14) withstood a maximum force of approximately 1.35 kg before rupture at around 15 seconds.

These findings confirm that the prepared film possesses adequate burst resistance to endure handling, encapsulation, and unfolding inside the gastric environment, while still allowing controlled rupture and expansion required for drug release. The burst strength results are consistent with the tensile strength and folding endurance data, together demonstrating that the formulation achieved a balanced mechanical profile suitable for gastroretentive applications.

While mechanical properties are not direct predictors of drug release kinetics, they are functionally important for ensuring that an expandable gastroretentive film can be handled, encapsulated, unfolded, and maintain structural integrity during hydration and gastric agitation. In this study, mechanical testing (folding endurance, tensile strength, and burst resistance) was performed primarily on the optimized formulation (F14) as a final confirmation that the selected candidate possesses sufficient robustness to withstand handling and in situ unfolding without cracking or premature rupture. Therefore, a formulation-by-formulation correlation analysis between mechanical parameters and dissolution/gastroretention outcomes cannot be robustly established within the current dataset. Nevertheless, the favorable mechanical profile of F14 is consistent with its observed in vitro integrity over 12 h and sustained release behavior, as inadequate mechanical integrity (e.g., brittleness or early rupture) would be expected to compromise the film’s structural persistence and potentially alter the release profile through increased fragmentation and surface-area changes.

### FT-IR spectrometry study

**Fig 9:**
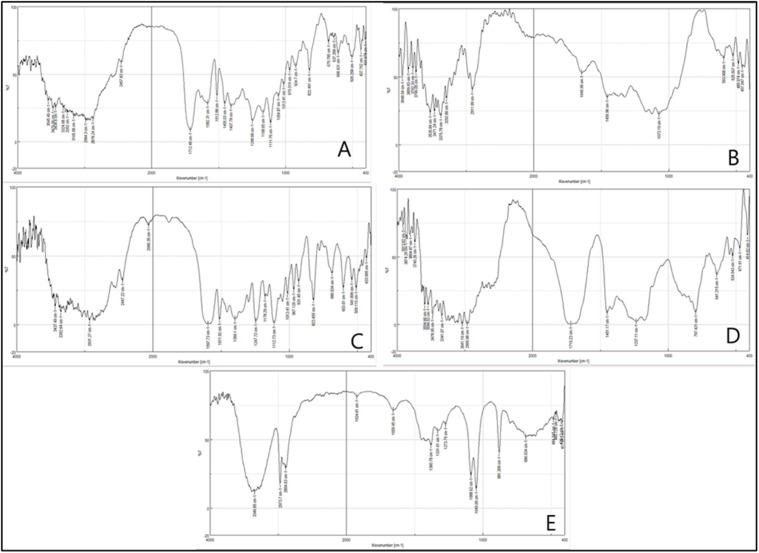
FT-IR spectra of: (A) Metoprolol, (B) Physical mixture, (C) Optimized formulation (F14), (D) HPMC, and (E) Carbopol 934p.

To examine potential interactions between Metoprolol and excipients, an FT-IR study was performed. The analysis was made for the pure drug sample, a physical mixture of the drug with polymers and other excipients, and the optimized formula (F14), and the results are shown in Fig 9. Comparing the spectra derived from the pure medicine with those from the physical combination, no loss of primary peaks associated with the drug was apparent. The spectra of F14 exhibited the presence of certain excipient peaks that overlap with the prominent peaks of the pure drug. FTIR spectrum of pure metoprolol tartrate exhibited distinct peaks in the vicinity of 3476 cm^−1^, 2984 cm^−1^, 2878 cm^−1^, and 2457 cm^−1^, which correspond to the stretching vibrations of −OH aliphatic and aromatic, NH_2_, NH, and –CH groups. Another notable peak was observed at 1582 cm^−1^, indicating the presence of a carboxylic acid salt. Additionally, an aromatic ring was identified based on the peak observed at 1512 cm^-1^. Nevertheless, certain peaks observed in the F14 film spectra showed good correlation with those of metoprolol (2894, 2973 cm-1). The O-H stretching peak is broadening in the (F14) spectrum, and this is primarily because of the hydrogen bond between the drug and the polymers [19]. The overlapping observed in the F14 spectra can be attributed to the drug being incorporated into the polymers’ matrices, and the absence of obvious peak shifts in the (F14) IR spectrum indicates that there is no possible interaction between metoprolol and the polymers used in the current work.

### XRD analysis

**Fig 10:**
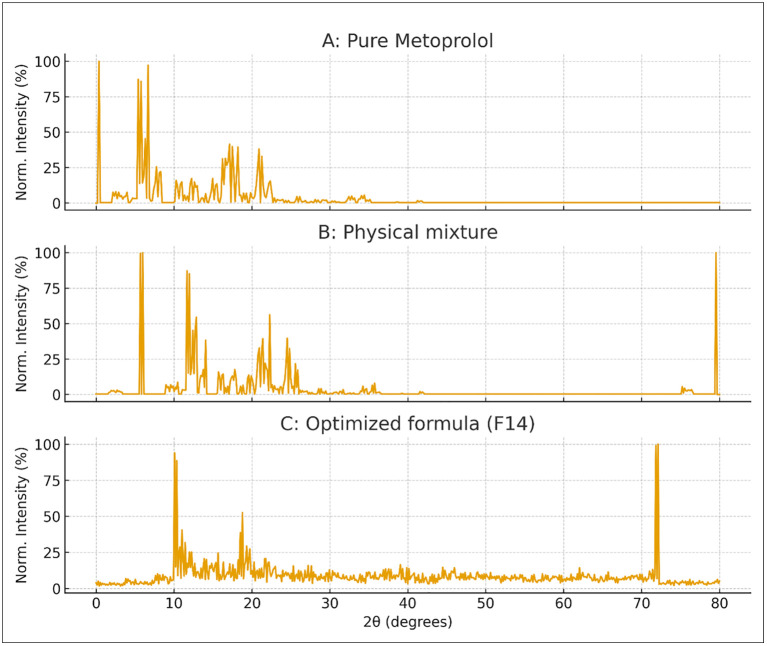
Normalized XRD patterns of (A) Pure Metoprolol, (B) Physical mixture, and (C) Optimized formulation (F14). Pure Metoprolol showed sharp peaks at 2θ = 14.8°, 18.9°, 22.2°, and 26.5°, confirming crystallinity. All diffractograms are shown in normalized intensity (0–100%) for direct comparison.

In X-ray diffraction (XRD) analysis, the 2θ values represent the diffraction angles that are characteristic of the crystalline structure of a compound, as defined by Bragg’s law. Each crystalline material produces distinctive diffraction peaks at specific 2θ positions, which serve as its structural fingerprint. In our study:

- Pure Metoprolol (Fig 10A): The diffractogram showed distinct, sharp crystalline peaks at 2θ ≈ 14.8°, 18.9°, 22.2°, and 26.5°, confirming the crystalline nature of the pure drug.
- Physical mixture (Fig 10B): These peaks were still observed but with lower intensity, indicating partial contribution from the crystalline drug diluted by excipients.
- Optimized formulation F14 (Fig 10C): The sharp peaks corresponding to the drug disappeared, and only a broad diffuse halo was observed. The absence of distinct peaks at the above 2θ positions confirms that the drug lost its crystalline structure and transformed into an amorphous form.

This transition from crystalline to amorphous state demonstrates the molecular dispersion of Metoprolol in the polymeric matrix, supporting its incorporation into the film and the potential for improved solubility and stability [19].

### SEM study

**Fig 11:**
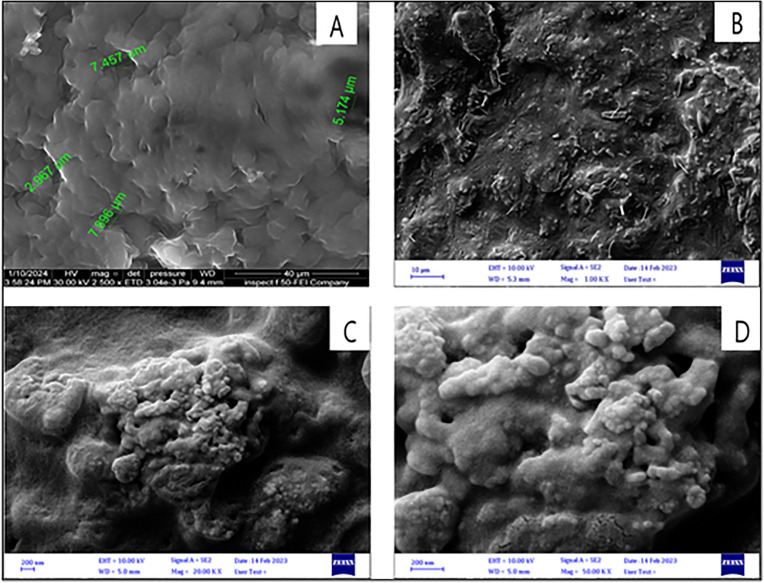
SEM Photograph of (A) Blank formula at Mag = 25.00 K X, (B) F14 at Mag = 1.00 K X, (C) F14 at Mag = 20.00 K X, and (D) F14 at Mag = 50.00 K X.

The SEM was conducted to examine the morphology of the optimized film, as seen in Fig 11B–D. The optimized film showed a rough surface with many pores presented on its surface, like the blank film, Fig 11A. This represents the success of the film prepared and the proper method used to prepare the gastroretentive films. Moreover, the rough surface indicates proper polymer incorporation, as no layer separation was observed in the film topography [32].

### DSC analysis

**Fig 12:**
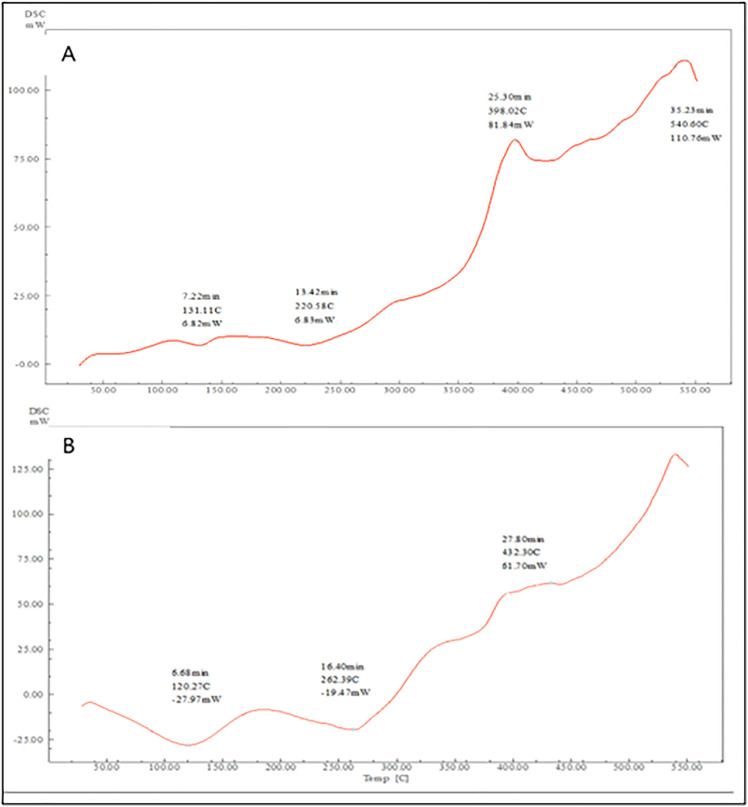
DSC Thermograms of (A) Physical mixture, and (B) F14.

DSC investigations were conducted to examine the drug's physical state throughout the film matrix. Comparing the melting point of the drug in the physical mixture to the optimized film (F14), it was evident that the melting point of the drug, which is 131.1 °C, is absent in the optimized film. According to Fig 12, the absence of the characteristic peak may indicate proper drug incorporation into the film's polymeric matrix [33].

### Film integrity

**Fig 13:**
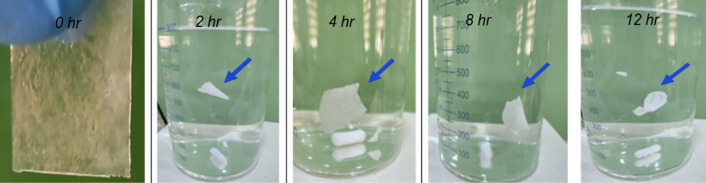
Integrity of the optimized gastroretentive film (F14) at different time intervals (0, 2, 4, 8, and 12 h) in 0.1 N HCl medium, showing its ability to maintain structural stability during the test period.

As shown in Fig 13, the film maintained its integrity for up to 12 hours in the dissolution medium, demonstrating strong mechanical stability and sustained drug-release potential. This performance is mainly attributed to HPMC, which swells to form a gel matrix that promotes flotation and protects the film from degradation, together with Carbopol, which enhances swelling and mucoadhesion [34,35].

### Radiographic evaluation

Radiographic imaging in this study was intended as a supportive, qualitative tool to verify gastric location at discrete timepoints rather than a comprehensive or quantitative assessment of gastroretention. While the presence of the radiopaque marker within the gastric region at 6 and 12 h suggests persistence of the dosage form in the stomach under the described conditions, radiography does not provide continuous residence-time tracking, nor does it fully characterize dynamic factors such as gastric motility, emptying patterns, or inter-individual variability. In addition, sedation and the limited sample size may influence gastrointestinal transit and limit extrapolation to other physiological conditions (e.g., the fed state).

**Fig 14:**
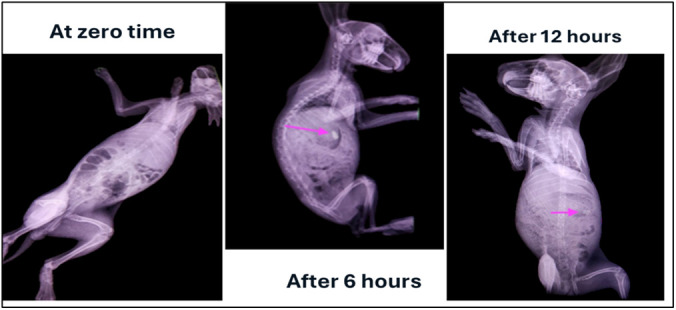
Radiographic images showing the gastric retention of the optimized formulation (F14) at different time intervals after administration, demonstrating its expansion and persistence in the stomach.

The optimized formula was administered orally to the rabbits, and the images were taken by X-ray at different predetermined time intervals. As illustrated in Fig 14, the formula remained swollen in the rabbit's stomach for 12 hr.

This extended retention period underscores the potential of the optimized formulation to maintain its integrity and functionality in a dynamic gastric environment [36].

A correlation between prolonged gastric retention and enhanced bioavailability of various drugs has been demonstrated previously. The findings of the present study complement this observation, showing that swelling-based gastroretentive films can sustain drug release, thereby ensuring stable plasma concentrations for up to 12 hours [37].

Although the use of radiographic imaging as a non-invasive and reliable method for tracking the location and behavior of gastroretentive systems *in vivo* has had a significant impact, the adoption of radiographic evaluation confirms the dosage form's functionality. It mirrors the methodology employed in these earlier investigations. However, this *in vivo* study serves as a preliminary evaluation to demonstrate the feasibility and potential of the developed film, and that future studies with a larger animal cohort would be required for robust statistical validation and quantitative assessment of gastric residence time.

Accordingly, the performance of F14 was compared with that of extended-release metoprolol dosage forms reported in the literature. For example, Issa *et al.* (2022) developed extended-release mini-tablets supported by design of experiments and physiologically based biopharmaceutics modelling, achieving ~80–90% release at 12 h under optimized conditions [38]. Similarly, Khobragade *et al.* (2022) formulated a multi-unit pellet system (MUPS) of metoprolol succinate that provided prolonged drug release with acceptable stability [39]. While these dosage forms demonstrated extended release, they rely primarily on conventional matrix or coating approaches and do not address gastric residence as a limiting factor for absorption. In contrast, our optimized expandable film (F14) achieved ~90% drug release at 12 h while simultaneously exhibiting significant swelling and in vivo gastric retention for 12 h, ensuring localized release at the primary absorption site. This dual-function sustained-release formulation, combined with prolonged gastric residence, offers a distinct advantage over conventional dosage forms by potentially enhancing bioavailability, reducing the need for frequent dosing, and improving therapeutic consistency.

### Stability study

In addition to confirming gastric retention *in vivo*, the short-term stability of the optimized film (F14) was further assessed under accelerated ICH conditions to evaluate its robustness during storage.

As summarized in Table 5, the optimized gastroretentive film (F14) demonstrated good stability under accelerated ICH conditions (40 °C/75% RH) over 6 weeks. Packed films retained clarity, flexibility, and smooth surfaces, with no cracking, discoloration, or tackiness. At the same time, unpacked samples became slightly tacky by week 3, reflecting the inherent humidity sensitivity of hydrophilic polymer matrices. This finding highlights the importance of protective packaging, as moisture uptake can plasticize HPMC/Carbopol networks and alter handling properties if unprotected [40,41].

**Table 5:** Short-term stability data of the optimized film (F14) stored under accelerated ICH conditions (40 ± 2 °C / 75 ± 5% RH) for 6 weeks (packed samples). Values are expressed as mean ± SD (n = 3).

Parameter	0 weeks	3 weeks	6 weeks
Thickness (µm)	152 ± 5.3	153 ± 5.2	154 ± 6.9
Weight (mg)	50.8 ± 1.2	51.0 ± 1.3	51.5 ± 1.5
Moisture content (% w/w)	4.3 ± 0.3	5.0 ± 0.4	5.8 ± 0.5
Tensile strength (MPa)	17.8 ± 1.3	17.0 ± 1.1	16.3 ± 1.2
Film burst (kg at 15 sec)	1.35 ± 0.17	1.32 ± 0.21	1.39 ± 0.09
Swelling index at 2 h (%)	138 ± 6.1	142 ± 7.8	147 ± 8.5
Cumulative release at 12 h (%)	86.2 ± 2.0	87.5 ± 2.2	88.9 ± 2.4

The stability of the formulation was further supported by solid-state characterization. FTIR spectra showed no new bands or major shifts, confirming the preservation of both excipient and drug functional groups and the absence of chemical interaction at 6 weeks. Together with the consistent release kinetics, these results indicate that the optimised film remained physically and chemically stable under short-term accelerated conditions.

## Conclusion

This work provides the first systematic demonstration of how polymer composition critically governs the design and performance of an expandable gastroretentive Metoprolol film. By applying a 4 × 4 full factorial design, 16 formulations (F1–F16) were generated and optimized. HPMC enhanced bioadhesion and facilitated drug diffusion, while Carbopol contributed to swelling and sustained release. The optimized formulation (F14), identified through multi-response optimization, exhibited desirable mechanical strength, rapid expansion upon hydration, and a controlled release profile extending up to 12 hours.

Complementary characterization supported these findings: FT-IR and XRD confirmed the amorphous dispersion of Metoprolol without drug–polymer interaction, kinetic modeling indicated non-Fickian (anomalous) release governed by both diffusion and polymer relaxation, and SEM analysis revealed a uniform film morphology with interconnected pores favoring sustained release.

Beyond vitro performance, F14 maintained its integrity *in vivo*, as confirmed by radiographic studies showing gastric retention for 12 hours. Short-term accelerated stability testing further validated its robustness, demonstrating that F14 retained its physical and chemical stability as well as its sustained-release characteristics over 6 weeks.

Taken together, these results underscore the novelty of F14, which uniquely combines significant swelling capacity, prolonged gastric retention, and sustained drug release in a single delivery platform. Such dual functionality is rarely achieved in conventional dosage forms, highlighting the potential of expandable gastroretentive films to improve therapeutic consistency and patient adherence. Future studies should extend this work through long-term stability assessments and comprehensive pharmacokinetic investigations to establish in vivo release behavior, bioavailability, and clinical applicability.

### Limitations of the study

The study was limited to in vivo radiographic evaluation in rabbits to confirm gastric retention and expansion of the optimized expandable film. No pharmacokinetic or bioavailability studies were performed; therefore, a direct relationship between prolonged gastric residence and systemic drug exposure could not be established. Additionally, radiographic imaging at discrete timepoints provided only qualitative confirmation of gastric location and did not capture continuous residence time, motility-related variability, or differences between fed and fasted states. These factors limit the quantitative assessment and generalizability of gastroretentive behavior.

### Future perspectives

Future work should include in vivo pharmacokinetic and bioavailability studies to quantitatively assess the effect of prolonged gastric retention on systemic exposure and therapeutic efficacy of metoprolol. Long-term stability studies under real-time ICH conditions are also warranted. Expanding in vivo evaluations to larger animal cohorts and human studies would enhance translational relevance. Furthermore, the expandable gastroretentive film platform may be investigated for other drugs with narrow absorption windows or short half-lives, highlighting its potential as a versatile gastroretentive delivery system.

## Supporting information

S1 Data DATASET. (XLSX)
